# Predictive nomogram model for major adverse kidney events within 30 days in sepsis patients with type 2 diabetes mellitus

**DOI:** 10.3389/fendo.2022.1024500

**Published:** 2022-12-16

**Authors:** Qi Xin, Tonghui Xie, Rui Chen, Hai Wang, Xing Zhang, Shufeng Wang, Chang Liu, Jingyao Zhang

**Affiliations:** ^1^ Department of Hepatobiliary Surgery, The First Affiliated Hospital of Xi’an Jiaotong University, Xi’an, Shaanxi, China; ^2^ Department of General Surgery, The First Affiliated Hospital of Xi’an Jiaotong University, Xi’an, Shaanxi, China; ^3^ Department of Surgical Intensive Care Unit, The First Affiliated Hospital of Xi’an Jiaotong University, Xi’an, Shaanxi, China

**Keywords:** sepsis, type 2 diabetes mellitus, major adverse kidney events within 30 days, nomogram model, early warning

## Abstract

**Background:**

In sepsis patients, Type 2 Diabetes Mellitus (T2DM) was associated with an increased risk of kidney injury. Furthermore, kidney damage is among the dangerous complications, with a high mortality rate in sepsis patients. However, the underlying predictive model on the prediction of major adverse kidney events within 30 days (MAKE30) in sepsis patients with T2DM has not been reported by any study.

**Methods:**

A total of 406 sepsis patients with T2DM were retrospectively enrolled and divided into a non-MAKE30 group (261 cases) and a MAKE30 group (145 cases). In sepsis patients with T2DM, univariate and multivariate logistic regression analyses were conducted to identify independent predictors of MAKE30. Based on the findings of multivariate logistic regression analysis, the corresponding nomogram was constructed. The nomogram was evaluated using the calibration curve, Receiver Operating Characteristic (ROC) curve, and decision curve analysis. A composite of death, new Renal Replacement Therapy (RRT), or Persistent Renal Dysfunction (PRD) comprised MAKE30. Finally, subgroup analyses of the nomogram for 30-day mortality, new RRT, and PRD were performed.

**Results:**

In sepsis patients with T2DM, Mean Arterial Pressure (MAP), Platelet (PLT), cystatin C, High-Density Lipoprotein (HDL), and apolipoprotein E (apoE) were independent predictors for MAKE30. According to the ROC curve, calibration curve, and decision curve analysis, the nomogram model based on those predictors had satisfactory discrimination (AUC = 0.916), good calibration, and clinical application. Additionally, in sepsis patients with T2DM, the nomogram model exhibited a high ability to predict the occurrence of 30-day mortality (AUC = 0.822), new RRT (AUC = 0.874), and PRD (AUC = 0.801).

**Conclusion:**

The nomogram model, which is available within 24 hours after admission, had a robust and accurate assessment for the MAKE30 occurrence, and it provided information to better manage sepsis patients with T2DM.

## Introduction

Sepsis is a life-threatening organ dysfunction due to a dysregulated host response to infection induced by bacterial, viral, or fungal infection (1). Sepsis is currently the leading cause of mortality for patients, accounting for over 10% of the mortality within hospitals (1, 2). Furthermore, sepsis is associated with a mortality rate of 25–30% (3). It was reported that between 17–20% of the patients with sepsis had diabetes mellitus (4, 5). With the wide adoption of Western food and lifestyles, it is projected that the prevalence of T2DM will exceed 700 million worldwide and may soon reach pandemic levels (6). Due to immune system dysfunction, diabetic patients have an increased propensity to develop infections and are at a higher risk for sepsis (2 - 6 times) (7, 8).

Additionally, sepsis is particularly harmful to the kidney, which makes sepsis-induced acute kidney injury (S-AKI) a risk factor for increased mortality rate (9). Moreover, a growing body of research has revealed that T2DM was associated with an increased risk of S-AKI, but did not increase the overall mortality of sepsis patients (5, 8, 10). The renal damage may be due to increased activation of NF-kappa B, TGF-β and oxidant levels as a result of consistent hyperglycaemia milieu, or it may result from end-organ damage by atherosclerosis (8). Nevertheless, S-AKI cannot be used to assess clinically major renal adverse events such as death, and dialysis dependency, among others. The National Institute of Diabetes and Digestive and Kidney Diseases workgroup on clinical trials in Acute Kidney Injury (AKI) recommended the use of MAKE30 as an endpoint in 2012 (11). Effects of the short-term or longer-term evolution of AKI were captured by MAKE30, a composite of death, new RRT, or PRD (11). Furthermore, MAKE30 was a composite and objective clinical outcome measure for sepsis patients that reflected comprehensive renal outcomes better than just a single complication, AKI. Therefore, early MAKE30 prediction and prompt personalized management may enhance the clinical prognosis.

It is crucial to select appropriate endpoints in clinical trials. Although there is consensus that serum creatinine and urine volume are employed as AKI predictors, the serum creatinine and urine volume have a significant variation until 50% of renal function is lost, indicating that none of them are particularly meaningful to patients. In addition, researchers are beginning to understand that patient-centered outcome, such as mortality, dialysis, and chronic kidney disease development, are more important for patients. With the help of MAKE30, it is possible to detect short-term effects on AKI, improve target therapy, and facilitate the conduct of clinical trials. The MAKE30 has been used as an adequate test endpoint in several clinical trials including the SALT, SMART, and pediatric sepsis trials (12–14). There are no trustworthy or reliable prediction models, however, to identify MAKE30 in sepsis patients with T2DM. We hypothesized that a nomogram model, based on routine biomarkers available within 24 hours of admission, may be of significant clinical value to predict MAKE30 because conventional biochemical indications are intrinsically unstable when used as a single index. To identify high-risk individuals likely to develop MAKE30 in sepsis patients with T2DM, the goal of our study was to identify the risk variables for MAKE30 and build an early prediction model.

## Materials and methods

### Study design

Between January 2015 and December 2021, 406 sepsis patients with T2DM participated in a retrospective cohort study (project number: 81773128), and anonymized clinical data are obtained from the Biobank of First Affiliated Hospital of Xi’an Jiaotong University (Xi’an, China).

### Patients

All sepsis patients with T2DM (18 years old and above) were evaluated for study enrollment. The sepsis 3.0 criteria were used for sepsis diagnosis (1). Furthermore, T2DM patients were identified if one of the following conditions was met: (1) self-reported diagnosis of T2D, (2) fasting plasma glucose (FPG) ≥7.0 mmol/L or (3) having received T2DM medications according to the 2022 American Diabetes Association (ADA) criteria (15). The following were the exclusion criteria: (1) below 18 years old; (2) hospitalization less than 24 hours; (3) A history of chronic kidney disease (stage 4-5) or renal transplantation, or current hemodialysis; (4) Hematological disorders. Participants were then divided into MAKE30 group and non-MAKE30 group based on their diagnostic criteria (16). Any one of the following criteria can be used to diagnose MAKE30 composite endpoints: (1) death; (2) receiving RRT for the first time; (3) a PRD (defined as a final inpatient serum creatinine value greater than or equal to 200% of baseline). These three components of MAKE30 were removed 30 days after inclusion or at hospital release, whichever came first. Furthermore, baseline serum creatinine was determined as follows: (1) If available, the lowest value was measured between 12 months and 24 hours prior to hospitalization; (2) When measured values were unavailable, an estimate was made using the formula previously described [creatinine(μmol/l)= 88.4 × (0.74 − 0.2 + 0.003 × age) in females, and creatinine (μmol/l) = 88.4 × (0.74 + 0.003 × age) in males] (16, 17).

### Data collection

The general information immediately available within 24 hours after admission included age, gender, temperature, Heart Rate (HR), Respiratory Rate (RR), MAP, source of infection, and Sequential Organ Failure Assessment (SOFA). The level of White Blood Cells (WBC), Neutrophil Percentage (NEUT%), lymphocyte, monocyte, PLT, Procalcitonin (PCT), Prothrombin Time Activity (PTA), Thrombin Time (TT), International Normalized Ratio (INR), Fibrinogen Degradation Products (FDP), D-Dimer (D-D), Fibrinogen (FIB), Activated Partial Thromboplastin Time (APTT), Prothrombin Time (PT), Globin (GLB); Albumin (ALB); Total Bilirubin (TBiL); Lipoprotein(a) (Lp(a)); apoE, apolipoprotein B (apoB), apolipoprotein A (apoA), Low-Density Lipoprotein (LDL), HDL, Triglycerides (TGs), Total Cholesterol (TC), urinary glucose, Uric Acid (UA), cystatin C, Blood Urea Nitrogen (BUN), Creatinine (Cr), and Total Cholesterol (TC) were also recorded within 24 hours after admission.

### Statistical analysis

Mean ± standard deviation was used to express the continuous variables that conformed to the normal distribution, median (interquartile range) was used to express non-normally distributed continuous variables, and categorical variables were expressed by the percentages. To further identify the independent predictors of MAKE30, univariate and multivariate logistic regression models were used. The corresponding nomogram was constructed using the output from multivariate logistic regression analysis, and then we constructed an online dynamic nomogram using the “DynNom” package. The ROC curves were used to evaluate the accuracy of independent predictors of MAKE30. The calibration curves were drawn to assess the consistency of the observed results and predicted probability. Decision Curve Analysis (DCA) was performed to assess the clinical net benefit of the predictive model. Finally, an analysis of the secondary outcomes using ROC curves was performed to assess the discrimination of the nomogram for 30-day mortality.

SPSS 26.0 software and R version 4.1.2 were used for the statistical analysis, at P < 0.05.

## Results

### Basic characteristics

**Figure 1:**
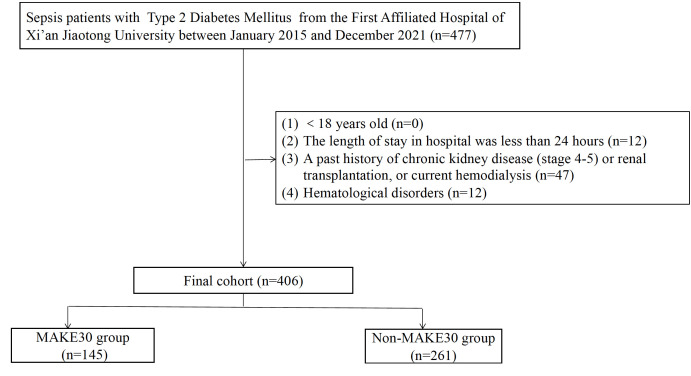
The flowchart of patient selection.

**Figure 2:**
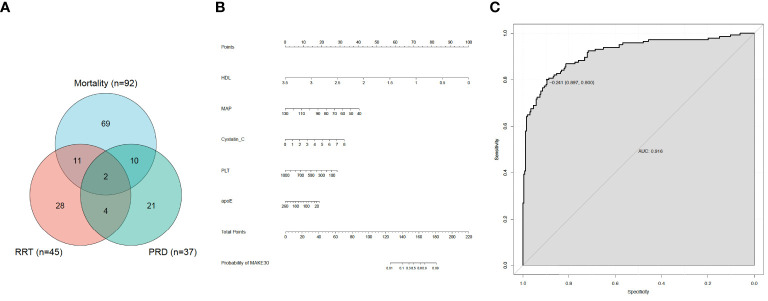
MAKE30 in sepsis patients with type 2 diabetes mellitus. **(A)** Venn diagram of major adverse kidney events within 30 days (MAKE30) components; **(B)** Nomogram to estimate the risk of MAKE30 in sepsis patients with type 2 diabetes mellitus; **(C)** The ROC curve of the nomogram for predicting MAKE30 in sepsis patients with type 2 diabetes mellitus.

In the final analyses, 406 sepsis patients with T2DM in total were selected (**Figure 1**). Of the 406, 145 (35.7%) patients reached MAKE30 during hospitalization. For the individual components, the mortality incidence was 92 (22.7%), new RRT was 45 (11.1%), and PRD was 37 (9.11%). Venn diagram shows the relationship among MAKE30 components more intuitively (**Figure 2A**). The median age of participants was 62 years (range, 57 to 71), with 39.2% of patients being female. Patients with MAKE30 were significantly older than those with non-MAKE30 (**Table 1**). Moreover, patients with MAKE30 had significantly higher SOFA scores than those with non-MAKE30 (10, 8-13 vs. 5, 3-7; P < 0.001). There were significant differences in the MAP (67, 63-90 vs. 93, 80-112; P < 0.001), HR, and infection sources between the MAKE30 group and the non-MAKE30 group. Nevertheless, gender, temperature, and RR were not significantly different at P > 0.05.

**Table 1:** The demographic and clinical data of sepsis patients with diabetes between the non-MAKE30 and MAKE30 groups.

Variables	Total (n=406)	Non-MAKE30 (n=261)	MAKE30 (n=145)	OR (95%CI)	*P* value
Age (years)	62 (51-71)	61 (49-71)	64 (54-71)	1.019 (1.005-1.034)	0.010
Female	159 (39.2%)	101 (38.7%)	58 (40.0%)	0.947 (0.625-1.434)	0.797
Temperature(°C)	36.7 (36.3-37.3)	36.7 (36.3-37.3)	36.6 (36.2-37.5)	1.136 (0.910-1.418)	0.259
HR (bpm)	93 (80-112)	89 (80-105)	97 (82-120)	1.014 (1.004-1.023)	0.004
RR (bpm)	20 (18-22)	20 (18-22)	21 (19-23)	1.018 (0.980-1.057)	0.357
MAP (mmHg)	88 (76-99)	91 (82-100)	67 (63-90)	0.915 (0.897-0.932)	<0.001
**Infection source**	0.005					
Pulmonary infection	94 (23.2%)	49 (18.8%)	45 (31.0%)		
Intra-abdominal infection	177 (43.6%)	131 (50.2%)	46 (31.7%)		
Urinary infection	59 (14.5%)	39 (14.9%)	20 (13.8%)		
Central nervous system infection	24 (5.9%)	14 (5.4%)	10 (6.9%)		
Skin and soft tissue infections	21 (5.2%)	13 (5.0%)	8 (5.5%)		
Cardiovascular system infections	31 (7.6%)	15 (5.7%)	16 (11.0%)		
SOFA	7(4-9)	5(3-7)	10(8-13)	2.000 (1.749-2.287)	<0.001

### Univariate analyses of clinical biochemical indicators

All the clinical biochemical indicators were available within 24 hours of admission as shown in **Table 2**. Univariate analyses revealed that the level of NEUT, PCT, TT, INR, FDP, D-D, APTT, PT, TBiL, UA, cystatin C (2.0, 1.1-2.9 vs. 1.1, 0.9-1.5; P < 0.001), BUN, and Cr significantly increased in the MAKE30 group compared with the non-MAKE30 group. On the contrary, the level of PLT (97, 58-151 vs. 162, 95-251; P < 0.001), PTA, apoE (35.5, 30.3-55.0 vs. 49.5, 34.8-75.2; P < 0.001), apoB, apoA, LDL, HDL (0.39, 0.29-0.51 vs. 0.70, 0.58-0.90; P < 0.001), and TGs were significantly lower in the S-AKI group, compared with the non-MAKE30 group.

**Table 2:** Univariate analyses of clinical biochemical indicators between non-MAKE30 and MAKE30 groups in sepsis patients with T2DM.

Variables	Total (n=406)	Non-MAKE30 (n=261)	MAKE30 (n=145)	OR (95%CI)	*P* value
WBC (x 10^9^/L)	10.4 (6.1-14.9)	10.2 (5.9-16.1)	10.9 (6.7-14.1)	0.985 (0.960-1.012)	0.279
NEUT (%)	86.3 (76.5-91.9)	85.5 (73.1-90.9)	89.2 (80.0-93.0)	1.021 (1.004-1.039)	0.017
Lymphocyte (x 10^9^/L)	0.75 (0.43-1.20)	0.77 (0.46-1.27)	0.70 (0.37-1.06)	0.838 (0.630-1.115)	0.225
Monocyte (x 10^9^/L)	0.39 (0.26-0.64)	0.39 (0.26-0.61)	0.45(0.24-0.69)	1.409 (0.797-2.491)	0.239
PLT (x 10^9^/L)	134 (85-209)	162 (95-251)	97 (58-151)	0.992 (0.990-0.995)	<0.001
PCT (ng/ml)	6.5 (1.1-23.0)	3.3 (0.59-17.7)	16.6 (3.9-35.72)	1.013 (1.007-1.020)	<0.001
PTA (%)	72.0 (54.0-87.5)	76.0 (60.0-89.1)	61.1 (44.5-83.4)	0.978 (0.968-0.987)	<0.001
TT (S)	14.7 (1.0-17.2)	14.6 (1.0-16.7)	15.5 (1.03-18.1)	1.011 (1.003-1.019)	0.009
INR	1.2 (1.1-1.4)	1.2 (1.1-1.4)	1.3 (1.1-1.6)	2.156 (1.353-3.436)	0.001
FDP (mg/L)	10.0 (4.5-22.4)	9.0 (4.3-19.1)	12.2 (5.8-30.3)	1.004 (0.999-1.008)	0.106
D-D (mg/L)	3.4 (1.7-7.7)	3.1(1.5-6.5)	4.3 (1.9-9.7)	1.024 (1.003-1.045)	0.025
FIB (g/L)	4.8 (3.3-6.0)	4.9 (3.4-6.1)	4.4 (3.1-6.0)	0.924 (0.840-1.018)	0.109
APTT (S)	39.7 (35.0-46.4)	39.0 (35.0-44.6)	42.2 (35.3-53.7)	1.024 (1.011-1.036)	<0.001
PT (S)	14.8 (13.3-16.8)	14.6 (13.4-16.2)	15.6 (13.1-18.3)	1.034 (1.003-1.066)	0.030
GLB	24.6 (20.3-18.5)	25.0 (20.6,28.4)	24.1 (19.7,29.1)	1.006 (0.979-1.032)	0.682
ALB	30.7 (25.1-39.9)	30.5 (25.4,39.0)	31.2 (24.8,42.4)	1.004 (0.987-1.021)	0.645
TBiL	17.8 (10.7-34.6)	17.0 (10.6-29.0)	18.4 (10.8,43.8)	1.003 (1.000-1.006)	0.024
Lp(a) (mg/L)	103 (35-258)	97 (28-258)	135 (41-223)	1.000 (0.999-1.001)	0.991
apoE (mg/L)	46.8 (33.4-72.3)	49.5 (34.8-75.2)	35.5 (30.3-55.0)	0.984 (0.977-0.991)	<0.001
apoB (g/L)	0.70 (0.52-0.89)	0.74 (0.57-0.94)	0.62 (0.40-0.78)	0.319 (0.156-0.654)	0.002
apoA (g/L)	0.73 (0.50-0.87)	0.77 (0.64-0.87)	0.52 (0.36-0.81)	0.072 (0.031-0.167)	<0.001
LDL (mmol/L)	1.36 (0.90-2.14)	1.58 (1.02-2.21)	1.02 (0.66-1.75)	0.489 (0.371-0.644)	<0.001
HDL (mmol/L)	0.62 (0.44-0.77)	0.70 (0.58-0.90)	0.39 (0.29-0.51)	0.002 (0.01-0.009)	<0.001
TGs (mmol/L)	1.89 (1.11-3.48)	1.79 (1.16-3.97)	1.89 (1.03-2.86)	0.938 (0.900-0.977)	0.002
TC (mmol/L)	2.81 (2.03-3.84)	2.82 (2.08-3.95)	2.69 (1.92-3.72)	0.898 (0.801-1.008)	0.068
Urinary glucose (mmol/L)	11.2 (7.9-15.3)	12.0 (8.1-15.4)	10.4 (7.2-15.2)	0.990 (0.962-1.018)	0.466
UA (umol/L)	317 (213-436)	281 (203-380)	386 (281-531)	1.003 (1.002-1.005)	<0.001
Cystatin C (mg/L)	1.2 (0.9-1.9)	1.1 (0.9-1.5)	2.0 (1.1-2.9)	3.018 (2.262-4.028)	<0.001
BUN (mmol/L)	8.7 (5.4-15.2)	7.8 (5.1-11.5)	12.1 (6.7-23.0)	1.116 (1.083-1.150)	<0.001
Cr (umol/L)	108 (59-239)	89 (55-235)	132 (72-320)	1.005 (1.003-1.007)	<0.001

### Independent predictors of MAKE30 and nomogram development

The significant different variables, including age, HR, MAP, NEUT, PLT, PCT, PTA, TT, INR, FDP, D-D, APTT, PT, TBiL, apoE, apoB, apoA, LDL, HDL, TGs, UA, BUN, cystatin C and Cr were used in multivariate logistic regression analyses. Then, it revealed that in sepsis patients with T2DM, MAP (0.928,0.906-0.950), PLT (0.995,0.992-0.999), HDL (0.009,0.002-0.036), apoE (0.988,0.979-0.997), and cystatin C (1.960,0.360-2.826) were independent predictors for MAKE30 (**Table 3**). Furthermore, a nomogram based on these traits was created to predict MAKE30 in sepsis patients with T2DM (**Figure 2B**). Moreover, we provided an online version of this nomogram using the “DynNom” package for widespread use by physicians and researchers (https://diabetes-s-aki.shinyapps.io/DynNomapp/).

**Table 3:** Multivariate logistic regression analyses of independent predictors for MAKE30 in sepsis patients with T2DM.

Variables	β	SE	Wald	P-value	OR (95% Cl)
MAP	-0.075	0.012	39.339	<0.001	0.928 (0.906-0.950)
PLT	-0.005	0.002	7.205	0.007	0.995 (0.992-0.999)
HDL	-4.766	0.737	41.770	<0.001	0.009(0.002-0.036)
apoE	-0.012	0.005	6.485	0.011	0.988(0.979-0.997)
Cystatin C	0.673	0.187	13.005	<0.001	1.960(1.360-2.826)
Constant	8.710	1.245	48.945	<0.001	—

MAP, Mean Arterial Pressure; PLT, platelet; HDL, High-Density Lipoprotein; apoE, apolipoprotein E.

### Verification of the prediction model

**Figure 3:**
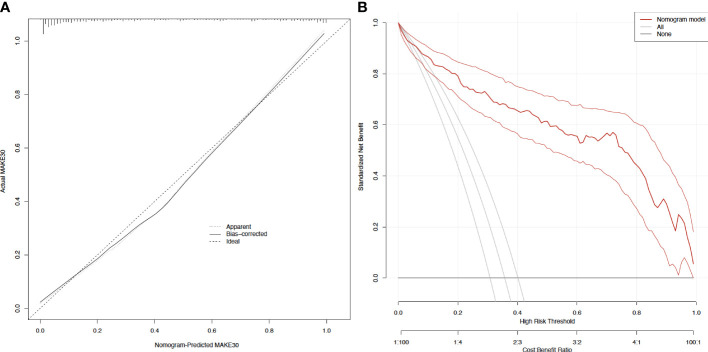
Calibration curves and decision curve analysis of the nomogram for predicting MAKE30 **(A)** Calibration curves of the nomogram; **(B)** Decision curve analysis of the nomogram.

In sepsis patients with T2DM, the diagnostic value of the nomogram model for MAKE30 was assessed using the ROC curve. The model had a good ability to predict MAKE30 (AUC = 0.916) as demonstrated in **Figure 2C**. Moreover, the predictive probabilities based on the calibration curve were consistent with the observation results, indicating a successful calibration (**Figure 3A**). In DCA, it was found that the nomogram has a superior overall net benefit within a wide and practical range of threshold probabilities, implying a high potential for clinical application (**Figure 3B**). Hence, in sepsis patients with T2DM, the nomogram model may be a robust MAKE30 predictor.

### The subgroup analyses using the prediction model

**Figure 4:**
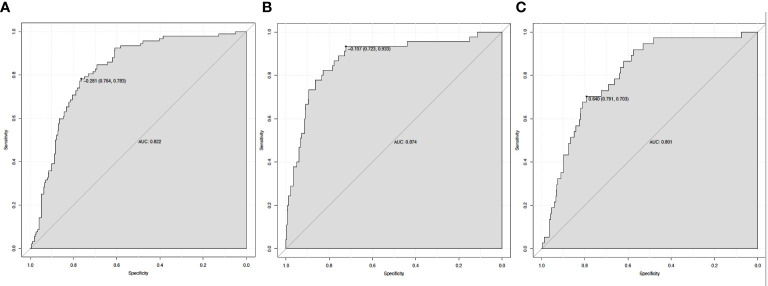
The ROC curve of the nomogram for predicting 30-day mortality **(A)**, new RRT **(B)**, and PRD **(C)**. RRT, renal replacement therapy; PRD, persistent renal dysfunction.

**Figure 4** illustrates how ROC curve analyses revealed that the nomogram model also had a high ability to predict the occurrence of 30-day mortality (AUC = 0.822), new RRT (AUC = 0.874), and PRD (AUC = 0.801) in sepsis patients with T2DM.

## Discussion

We constructed a simple nomogram model to predict MAKE30 in sepsis patients with T2DM based on those independent predictors (MAP, PLT, Cystatin C, HDL, and apoE) within 24 hours of admission. The most important thing was that the model worked well and was simple to apply. In addition, doctors must use a quick and precise nomogram model to anticipate MAKE30 to make tailored management decisions, which will improve the outcome and reduce mortality in sepsis patients with T2DM. Since sepsis is one of the most common causes of kidney injury, it is a prominent clinical problem in critically ill patients (18). Furthermore, more sepsis patients with T2DM 145 (35.7%) reached a MAKE30 composite outcome, than patients with acute pancreatitis (16%) and sepsis patients (28.3%) (16, 17).

The classical theories suggest that the primary pathogenic mechanisms causing kidney damage include decreased renal blood flow, secondary tubular epithelial cell death, or acute tubular necrosis. In the current study, sepsis with T2DM and a low level of MAP is an independent risk factor for MAKE30, which indicates renal tissue ischemia. However, more studies have revealed that various mechanisms, such as inflammation, microcirculatory dysfunction, and metabolic reprogramming, contribute to the pathogenesis of kidney injury, and that hemodynamic instability was not a necessary pathogenetic factor for kidney injury (19, 20). It is well known that during the sepsis period, inflammatory mediators are released in the intravascular compartment, causing damage to the vascular endothelium. Then, the coagulation system subsequently hyperactivated as a result of damaged vascular endothelium activating platelets first, which significantly caused the subsequent formation of micro-thrombi contributing to kidney injury (20, 21). Intimate and complex relationships existed between the inflammatory response and coagulation failure, both of which have a significant impact on the etiology of kidney damage in sepsis patients with type 2 diabetes. We also found that PLT was an independent predictor of MAKE30. Previous studies have demonstrated shown that PLT is important for controlling hemostasis as well as for interacting with other immune cells to modulate the immune and inflammatory response (22). Lv et al. also revealed that platelets contributed to kidney injury by inducing renal cell apoptosis (23).

In addition, we demonstrated that in sepsis patients with T2DM, cystatin C was an independent predictor of MAKE30. Protease inhibitors belonging to the cystatin superfamily, serum cystatin C, can pass freely through the glomerular filtration and then be completely reabsorbed and degraded by the renal tubules (24). It is not significantly influenced by biological factors such as age, gender, muscle mass, diet, infection, and tumors, in contrast to Creatinine (Cr). Serum Cr may not be able to detect very early changes in renal function, particularly in sepsis patients with early tubular necrosis (18). Nevertheless, serum cystatin C was able to predict the occurrence of AKI, one to two days earlier than serum Cr (18, 25). Cystatin C has been suggested as a potential predictor for the early diagnosis of S-AKI or was an alternative to the gold standard “creatinine” in growing number of studies in recent years (18, 26). Regardless of the underlying precipitating factors, previous studies have demonstrated that cystatin C played a significant role in the diagnosis and prediction of kidney injury in sepsis or other critical clinical conditions (25, 27). Moreover, in critically ill neonates, cystatin C could be used as a powerful predictor of kidney injury (28). When it was immediately estimated within 24 hours after admission for early detection of kidney injury, hence, serum or urine, cystatin C was very valuable (26).In addition, it was found that serum cystatin C was associated with the recovery, death, or renal replacement therapy of kidney injury (27, 29).

Future interventions may be directed at altering the apolipoproteins and cholesterol of sepsis patients, which was revealed by omic methods (30, 31). Proinflammatory cytokines can also alter the hepatic synthesis of apolipoprotein and acute phase reactants in the liver, such as tumor necrosis factor-alpha (TNF-α), interleukin-6 (IL-6), and interleukin-1 (IL-1) (32). This study revealed that apoE was an independent predictor for MAKE30. Increasing evidence has demonstrated that apoE is important in the pathophysiological course of anti-infection and anti-inflammation like sepsis (31). Previous studies have demonstrated that apoE 3 could protect porcine proximal tubular cells from gentamicin-induced injury (33), and apoE ϵ4 was protective against the development of kidney injury (34).

Decreased HDL concentrations are commonplace during an acute sepsis episode and are proportional to the degree of inflammation (32). Studies have shown that HDL levels decreased by 40–70% throughout the inflammatory process, which resulted in patients with sepsis having a bad prognosis (35). Furthermore, in patients with sepsis, reduced HDL levels were independently associated with an increased risk of kidney injury onset and decreased Glomerular Filtration Rate (GFR) (36). Chien et al. indicated that to avoid disease progression, multi-organ dysfunction, and renal injury in sepsis, HDL levels were a prognostic factor for making a personalized management plan (37). Reduced cholesterol may be the outcome of elevated erythropoietin brought on by hypoxia signaling activation in patients with ischemia-induced kidney impairment (38). Since kidneys are significant in the recycling of senescent HDL particles, clinical studies have demonstrated that low levels of HDL were associated with increased risk of kidney injury (35). Moreover, polymorphisms in HDL metabolism genes such as rs1800777 (allele A) in the CETP gene, were strongly associated with an increased risk of kidney injury during sepsis (39). Increased HDL levels may activate the endothelial nitric oxide synthase (eNOS) pathway, reducing the production of adhesion molecules, leucocytes activation, and neutrophil infiltration, reducing vascular impairment and renal parenchymal damage (35). The loss of HDL and its components is influenced by renal tubular injury influences tubular reabsorption function and catabolism (35). Extra renal synthesis and metabolism of HDL components can also be influenced by kidney injury (40). Therefore, in sepsis patients with T2DM, lower HDL is significantly associated with MAKE30 prediction

In this study, we developed a simple nomogram model (based on MAP, PLT, Cystatin C, HDL, and apoE) to predict MAKE30 in sepsis patients with T2DM. After verification, it demonstrated good performance in discrimination, calibration, and clinical application. This nomogram can also be used to determine the appropriate treatment options for high-risk patients. The application of the nomogram model is demonstrated by the following example: assuming a sepsis patient with T2DM with a MAP of 70 mmHg, a Cystatin C of 3 mg/L, an HDL of 1 mmol/L, a PLT of 200 x 10^9^/L, and an apoE of 40 mg/L. The score assigned to each parameter on the “Points” axis is obtained as shown in **Figure 2B**. The sum of points for each parameter is used to calculate the final score [27 (MAP) + 12 (Cystatin C) + 72 (HDL) + 23 (PLT) + 15 (apoE) = 149]. This score corresponds to about 33% risk of developing MAKE30. Another option is to use the online version (https://diabetes-s-aki.shinyapps.io/DynNomapp/) to obtain the same result easily. Finally, we also discovered that in sepsis patients with T2DM, the nomogram model had perfect predictive power for predicting 30-day mortality (AUC = 0.822), new RRT (AUC = 0.874), and PRD (AUC = 0.801).

### Limitations

Nevertheless, this study had some limitations. Firstly, since it was a single-center study, there was a chance of selection bias influencing the results. Secondly, it was a seven-year retrospective study, so there was significant advancement in the management decision-making process, affecting sepsis development. Thirdly, the model was constructed from a training group with a significantly smaller testing sample. Therefore, additional multi-center prospective studies with an adequate cohort size would be needed to assess its potential and validate the results.

## Conclusions

In conclusion, our study demonstrated that in sepsis patients with T2DM, the levels of MAP, PLT, Cystatin C, HDL, and apoE available within 24 hours after admission played a critical role in MAKE30 prediction. Moreover, the predictive nomogram model based on those predictors performed well in the discrimination, calibration, and clinical application for MAKE30, which is crucial for clinicians to make timely personalized management decisions.

## Data availability statement

The original contributions presented in the study are included in the article/**Supplementary Material**. Further inquiries can be directed to the corresponding authors.

## Ethics statement

This study was conducted following the Declaration of Helsinki and was approved by the Ethical Committee of the First Affiliated Hospital of Xi’an Jiaotong University. All patient data were analyzed in anonymity. Patient consent was waived by the ethics committee, as no individual data were published, nor was any intervention performed on patients.

## Author contributions

QX conceived of the study and drafted the manuscript. TX, RC, and XZ participated in the statistical analysis. HW participated the design of the study. SW, CL, and JZ participated in its design and coordination and helped to draft the manuscript. All authors contributed to the article and approved the submitted version.
